# Exposure Assessment in Cohort Studies of Childhood Asthma

**DOI:** 10.1289/ehp.1002267

**Published:** 2010-11-16

**Authors:** Victoria H. Arrandale, Michael Brauer, Jeffrey R. Brook, Bert Brunekreef, Diane R. Gold, Stephanie J. London, J. David Miller, Halûk Özkaynak, Nola M. Ries, Malcolm R. Sears, Frances S. Silverman, Tim K. Takaro

**Affiliations:** 1 Dalla Lana School of Public Health, Gage Occupational and Environmental Health Unit, University of Toronto, Toronto, Ontario, Canada; 2 School of Environmental Health, University of British Columbia, Vancouver, British Columbia, Canada; 3 Environment Canada, Air Quality Research Division, Toronto, Ontario, Canada; 4 Institute for Risk Assessment Sciences, Division of Environmental Epidemiology, Utrecht University, Utrecht, the Netherlands; 5 Department of Environmental Health, Harvard School of Public Health, Boston, Massachusetts, USA; 6 National Institute of Environmental Health Sciences, National Institutes of Health, Department of Health and Human Services, Research Triangle Park, North Carolina, USA; 7 College of Natural Sciences, Carleton University, Ottawa, Ontario, Canada; 8 U.S. Environmental Protection Agency, Research Triangle Park, North Carolina, USA; 9 Health Law Institute, University of Alberta, Edmonton, Alberta, Canada, Faculty of Law and School of Health Information Science, University of Victoria, Victoria, British Columbia, Canada; 10 Firestone Institute for Respiratory Health, McMaster University, Hamilton, Ontario, Canada; 11 Faculty of Health Sciences, Simon Fraser University, Burnaby, British Columbia, Canada

**Keywords:** childhood asthma, cohort studies, exposure assessment

## Abstract

**Background:**

The environment is suspected to play an important role in the development of childhood asthma. Cohort studies are a powerful observational design for studying exposure–response relationships, but their power depends in part upon the accuracy of the exposure assessment.

**Objective:**

The purpose of this paper is to summarize and discuss issues that make accurate exposure assessment a challenge and to suggest strategies for improving exposure assessment in longitudinal cohort studies of childhood asthma and allergies.

**Data synthesis:**

Exposures of interest need to be prioritized, because a single study cannot measure all potentially relevant exposures. Hypotheses need to be based on proposed mechanisms, critical time windows for effects, prior knowledge of physical, physiologic, and immunologic development, as well as genetic pathways potentially influenced by the exposures. Modifiable exposures are most important from the public health perspective. Given the interest in evaluating gene–environment interactions, large cohort sizes are required, and planning for data pooling across independent studies is critical. Collection of additional samples, possibly through subject participation, will permit secondary analyses. Models combining air quality, environmental, and dose data provide exposure estimates across large cohorts but can still be improved.

**Conclusions:**

Exposure is best characterized through a combination of information sources. Improving exposure assessment is critical for reducing measurement error and increasing power, which increase confidence in characterization of children at risk, leading to improved health outcomes.

There is a growing interest in advancing our understanding of environmental exposures that influence the development of disease. Asthma is a complex and heterogeneous syndrome, with a variable phenotype characterized by chronic airway inflammation, reversible airflow limitation, airway hyperreactivity, and excess mucous secretion (Bosse and Hudson 2007; von Mutius 2008). Multiple genes have been associated with the development of asthma (Ober and Hoffjan 2006), as have numerous exposures (Miller and Ho 2008) [see Supplemental Material, Table 1 (doi:10.1289/ehp.1002267)].

The environment clearly influences the development of allergies and asthma, because having a particular gene or combination of genes does not guarantee the development of these conditions. Occupational asthma likewise points toward the role of environment in disease development (Bernstein et al. 2006), as do the changes in asthma prevalence in the past 30–40 years (Platts-Mills et al. 2005; von Mutius 1998).

Gene and environment interactions are also suspected in asthma and allergies (Hunter 2005), as are epigenetic mechanisms (Baccarelli et al. 2009; Nawrot and Adcock 2009). However, knowledge about how environmental exposures participate in epigenetic mechanisms is in its infancy (Baccarelli et al. 2009; Miller and Ho 2008; Nawrot and Adcock 2009).

Longitudinal cohort studies are the most powerful observational study design for studying exposure–response relationships pertaining to disease development. Because of the relatively long observation period for cohort studies, it is also possible to undertake intervention studies to investigate selective avoidance and disease outcomes (Clayton and McKeigue 2001). Examples of this approach can be found in the Prevention and Incidence of Asthma and Mite Allergy study (PIAMA) (Brunekreef et al. 2002) as well as the Canadian Allergy Primary Prevention Study (CAPPS) (Becker et al. 2004).

Cohort studies have drawbacks: They are expensive and time consuming, and investigators must carefully plan the exposure assessment component (Rothman and Greenland 1998). Researchers must think critically about what can be measured, when to measure it, and the associated costs, because improved exposure assessment will lead to decreased measurement error and significantly increased study power (Armstrong 1996; London 2007; Wong et al. 2003).

Knowledge about which environmental exposures and behaviors confer risk is important because many environmental exposures are modifiable and thus preventable. Understanding the biological mechanisms that underlie the development of disease is necessary for the design of effective environmental interventions and therapeutics. The identification of causal exposures depends on accurate measurement of both exposures and outcomes; this is a challenge for many reasons, summarized in Supplemental Material, Table 2 (doi:10.1289/ehp.1002267).

Quantifying environmental exposures in cohort studies is complex and involves difficult decisions, including tradeoffs in the short and long term. Researchers designing cohort studies of childhood asthma must prioritize their research questions and refine exposure assessment strategy to ensure that their study makes a meaningful contribution.

## Objectives

This manuscript was motivated as a result of a workshop held in Banff, Canada, 9–10 February 2008, in connection with the launch of the Canadian Healthy Infant Longitudinal Development (CHILD) birth cohort (Subbarao et al. 2009). The purpose of this review is to discuss the design of environmental exposure assessment in longitudinal cohort studies of childhood asthma and allergies. This review is not intended to be a systematic review of the topics presented, but rather a focused discussion of, and a starting point for developing, more effective exposure assessment strategies pertaining to the environmental causes of asthma and allergy. The references cited herein guide readers to more in-depth discussions on specific issues raised, whereas this review as a whole provides a broader perspective on decisions that affect the environmental exposure data and ultimately the power of the cohort study under design. This review thus serves as an introduction to the challenges in exposure assessment that environmental epidemiologists continue to face.

## Results

### Developing an exposure assessment strategy

The exposures linked with childhood asthma in Table 1 [Supplemental Material (doi:10.1289/ehp.1002267)] are numerous and include those that may act through different uptake routes: inhalation, ingestion, and dermal. Historically, the research focus has been on inhalation, but this is changing, particularly with respect to occupational asthma (Redlich and Herrick 2008). Relevant exposures include biological, chemical, and physiological agents that can be encountered in a variety of environments including the home, school, and outdoors, and those that can start *in utero*. Although not discussed in detail here, parental take-home exposures related to their occupation, or paraoccupational exposures, may also play a role in the development of childhood asthma (Magnusson et al. 2006). Furthermore, as adolescents enter the workforce, they may have their own relevant occupational exposures (Syamlal and Mazurek 2008).

Three general strategies for characterizing environmental exposures include questionnaires, environmental samples, and predictive models. Measurement of biomarkers of exposure can also be used, but they are typically not applied in large cohort studies for asthma/allergies and therefore are not included in this review.

Questionnaires are a common tool for assessing exposure. Questionnaire data are typically surrogate measures for the exposure(s) of interest. The researcher relies on a conceptual model of how the surrogate is related to the exposure of interest and of how well the surrogate differentiates between different exposure levels.

Information on a variety of exposure and exposure scenarios, including inhalation and ingestion of biological and chemical agents, can be acquired from questionnaires [Supplemental Material, Table 3 (doi:10.1289/ehp.1002267)]. The main limitation of using questionnaires to assess exposure is recall bias (Le Moual et al. 2000). There are also limits to how much can be asked on a single questionnaire, how frequently it can be administered, and how specific it can be made. In addition, asking subjects about specific exposures on questionnaires assumes that the subject will recognize both the exposure agents and their own exposure. These issues can result in the wrong question or too many questions being asked, leading to imprecise data, subject fatigue, and/or a poor response.

Environmental samples or measurements are considered the most precise way to quantify the exposure of an individual at a point in time. Many exposures of interest can be measured, either directly or through collection of samples for subsequent analysis, at least for a short time period. However, whether measurements are practical with respect to the cost and the burden on subjects differs by exposure. In reality, a single measurement fails to describe the variability of an individual’s exposure over time, and the quantity of a specific agent measured in a child’s environment is, at best, a surrogate for dose.

Before deciding to undertake exposure measurements, researchers should consider whether a reliable and validated surrogate could be obtained more easily. Of particular importance is the type of data that will be generated from an environmental measurement versus a questionnaire. In some cases an exposure measurement may be unnecessary. For example, the presence of pets can be ascertained from questionnaires or from observation. But to determine the exposure–response relationship between pet dander and asthma symptoms, a measurement of cat dander in house dust is more desirable, because many houses without a furry pet have measurable levels of dander (Chew et al. 1998; Gehring et al. 2004; Nafstad et al. 2001). Similarly, recent research on thirdhand smoking indicates that residual tobacco smoke contamination remains after the cigarette is extinguished (Winickoff et al. 2009). Although exposures related to first- and secondhand smoking are believed to be assessed reliably through questionnaires, this additional route for tobacco product exposure may require modification to questionnaires or more emphasis on measurements (i.e., air sampling or urine cotinine) to develop sufficiently informative exposure measures.

In studies of the environment and childhood asthma, the exposures that tend to be measured quantitatively are indoor dust, house dust mite allergens, pet allergens, indoor air pollutants, and outdoor air pollutants. Many birth cohorts focusing on environmental determinants of childhood asthma and allergies have measured and reported these exposures at multiple time points and locations within the child’s environment. Table 1 summarizes the exposure assessment in a selection of large cohorts that focused on the development of childhood asthma in relation to environmental exposures other than air pollution. Table 1 highlights not only the similarities in exposures selected for measurement, but also the differences in timing and location of sample collection. These differences between studies complicate interpretation and comparison of results as well as subsequent data-pooling efforts.

The reliability of exposure measurements is limited by several factors, including the choice of exposure measured, how it relates to the relevant dose and/or biological pathway, and the method used to collect and analyze the sample, as well as the uncharacterized variability in exposure over time and place. Each will contribute to the overall error and variability of the measured value.

Investigators rarely, if ever, measure the actual exposure that an individual receives; ultimately, exposures are quantified based on surrogate information and a model (or a combination of models) that link this information to exposure based on common assumptions. The model can be simple, such as the presence of a furry pet equals exposure to dander, or more complex—using monitoring data for assigning air pollution exposure. In both cases, surrogates are measured, and an underlying model relates the surrogate to the exposure of interest.

### Estimating exposure using models: an air pollution example

In previous cohort studies of air pollution and childhood asthma, indicator variables or interpolation methods have been used to assign exposure based on a single piece of information [i.e., distance to major roads (Brunekreef et al. 1997)]. In recent years, complex modeling approaches have become common for assigning exposure. Approaches for modeling air pollutants vary; some use emissions or monitoring data as inputs, whereas others use measurements collected explicitly for the purposes of constructing the model (Brauer et al. 2003).

Land-use regression (LUR) models relate measurement data at multiple sites with readily available spatial information (from geographic information systems, such as road and pollutant emission location, to calculate air concentrations (i.e., estimated exposures) across an unsampled area with high resolution (50 m or better) (Brauer et al. 2003; Hoek et al. 2008). Regional-scale air quality models, such as the grid-based Community Multiscale Air Quality (CMAQ), use emissions and meteorologic inputs combined with knowledge on atmospheric chemistry, thermodynamics, and physics to provide temporally resolved predictions across large spatial areas (~ 1 × 1 km and larger grids) (Byun and Schere 2006). Local-scale air dispersion models (e.g., AERMOD) use emissions data to estimate local concentrations with smaller-scale spatial variability (Cimorelli et al. 2005). Unlike the regional-scale models, these do not incorporate detailed atmospheric chemical reactions, and meteorological information (e.g., wind direction and speed) is rarely available for all the locations of interest. Combining grid-based regional-scale and local-scale dispersion models is a current area of development to improve air quality modeling for exposure estimation.

A major limitation of outdoor air pollution information (modeled or measured) is that it does not account for the majority of time people spend indoors (Ozkaynak et al. 2008). Additional models, which combine air quality models and/or measurements with exposure models [Hazardous Air Pollutant Exposure Model (HAPEM)] and dose models [Stochastic Human Exposure and Dose Simulation (SHEDS)], are available. For example, SHEDS estimates time–activity patterns as well as microenvironment exposure differences for populations based on census data and time–activity studies (Burke et al. 2001; Isakov et al. 2009; Ozkaynak et al. 2009). Models can also be combined with time–activity information so that the movement of a subject over the course of the day and the resulting variation in exposure between outdoor microenvironments is accounted for (Nethery et al. 2009; Ryan et al. 2008). To apply these approaches to a cohort study requires subject-specific time–activity information, which is difficult to obtain for long time periods and thus tends to rely on snapshots of information from questionnaires.

The amount of detail and temporal resolution that can be obtained regarding exposure over the course of a cohort study is limited. It is impossible to evaluate all exposures continuously, and there are limitations to the measurement collection. Environmental exposure measures are usually time-averaged environmental concentrations relevant to the external exposure of the agent rather then the internal, or absorbed, dose. Furthermore, for some agents, cost-effective sampling and analytical methods are not available. For others it is a struggle to convert questionnaire responses into meaningful exposure metrics because of limited validation information.

### Prioritizing exposure assessment

There are too many exposures associated, or hypothesized to be associated, with childhood asthma to evaluate them all in a single study. Given limited resources, there are tradeoffs between number of exposures to explore and the accuracy and resolution with which they can be characterized. Additionally, no single exposure is likely to hold the key to childhood asthma [i.e., mono-interventions are ineffective (Maas et al. 2009)], and new hypotheses about environmental exposures will evolve over the course of a cohort study.

How should exposures be prioritized in the context of cohort studies? How many exposures should be studied? At what time point(s) or life stage will exposure be measured? How often within each life stage will exposure be measured? Will the exposure be constant or vary over the exposure window of interest? Can exposures with similar mechanisms be combined in analyses? These questions are critical to the design of a rigorous exposure assessment strategy. Examples of some general strategies that could help prioritize the exposures of interest in a cohort study are importance for public health, existing measurement capability, biological mechanisms, and potential for future data pooling. This list is not exhaustive, nor would strategies need to be employed exclusively.

When considering the public health importance of an exposure, priority should be given to exposures that are modifiable and prevalent in the population and have been hypothesized to be associated with development of asthma. Under this scheme, exposures of priority might be environmental tobacco smoke, building moisture levels, microflora related to building moisture, air pollution, and pest allergens (e.g. mite, cockroach, rodent). Exposures of secondary priority under this scheme might include those that are not easily or justifiably modifiable (e.g., pollen) as well as exposures that are highly speculative (e.g., diet, gut microflora, and phthalates). Modifiable exposures could occur at many levels, from the individual level (e.g., smoking) to the policy level (e.g., local policy on smoking in public areas, or national policy on air pollutants). The ability to reliably characterize the exposures of interest is a critical consideration when prioritizing exposures. The existence of a validated measurement method often influences the allocation of resources necessary to collect the desired data. In the case of ambient air pollutants, if a model has been developed for the study area, its application to derive exposure estimates for study participants is straight forward and low cost. However, if this model has not been developed, another method of estimating exposure may be more practical. Similarly, the validity and reliability of questionnaire items should also be considered if self-reported exposure data are to be collected. The validity and reliability of new questionnaire items can always be tested in a subset of participants, but this requires resources and could delay progress.

It is important to maximize the amount of information that can be retrieved from a single sample. Both dust samples and questionnaires can provide a significant amount of information on multiple exposures from a single sample. Dust samples provide the opportunity for quantifying exposure to a variety of agents (e.g., allergens, phthalates, and fungal markers) and when collected from a reservoir, such as deep carpet, can represent a time-integrated exposure (Roberts et al. 2009). Previous studies have also demonstrated that it is possible to have subjects participate in the collection of dust samples, making it more cost effective to collect multiple samples over time (Arbes et al. 2005; Schram-Bijkerk et al. 2006).

The biological mechanism of action can also be used as a method for prioritizing exposures. This can be conceptualized in two ways. First, exposures that act through the same biological pathway as other exposures that have been associated with asthma may be high priority for further study. Alternatively, if several exposures act through the same biological pathway, methods for quantifying biomarkers of exposure (or effect) within the pathway can be developed to measure dysfunction in the pathway. This dysfunction can then be related to the outcome or the exposure of interest, reducing exposure assessment costs.

Both of these approaches require the identification of pathways and mechanisms relevant to childhood asthma. We suggest five pathways, as a starting point, through which exposures could act to ultimately cause childhood asthma:

- Oxidative stress (Bhalla et al. 2009; Ciencewicki et al. 2008; MacNee 2001; Yang et al. 2008)
- Disruption of epithelial barrier function (Bhalla et al. 2009; Hammad and Lambrecht 2008; Holgate 2008; Knight and Holgate 2003)
- Adaptive versus innate immune response (Bhalla et al. 2009; Hammad and Lambrecht 2008)
- Disruption of normal airway development or later airway remodeling (Becklake and Kauffmann 1999; Folli et al. 2008; Postma 2007)
- Genetic and epigenetic inheritance (Bosse and Hudson 2007; Moffatt 2008; Ober and Hoffjan 2006; Yang et al. 2008).

These pathways are not mutually exclusive; often exposures (genetic or environmental) act through multiple pathways simultaneously, and pathways may also interact to cause detrimental effects in the lung (i.e., genetic control of immune response).

Interest in gene–environment interactions has created a need for studies with large sample sizes. Obtaining sufficient power to find a significant interaction is unlikely in any one cohort (because of small sample size) but is more likely with a meta-analysis or pooled analysis of multiple cohorts (Hunter 2005). These approaches could be particularly useful in genomewide association studies (GWAS) (Zeggini and Ioannidis 2009).

To successfully pool data across studies, it is ideal to plan for data pooling during study design. Ideally the exposure assessment strategy should be the same across all studies to be pooled. In reality, this means the same exposures are measured at the same time points using the same methods/protocols, which is difficult to achieve. To the extent that it is scientifically justified, new studies should consider harmonizing some target exposures with existing studies.

Power for a gene-by-environment study depends on four factors: the true effect size, the exposure prevalence, the genotype prevalence, and measurement error (Wong et al. 2003). It is not possible to manipulate exposure prevalence or genotype prevalence, but measurement error can be directly influenced by study design (Vineis 2004; Wong et al. 2003).

Measurement error can be reduced through refined research questions, accurate genotyping, improved understanding of the mechanism of effect, repeated measurements of exposure, and improved timing of exposure measurements (Vineis 2004; Wong et al. 2003). Improved exposure assessment will lead to more precise exposure estimates as well as decreased measurement error and significantly increased study power (Armstrong 1996; London 2007; Wong et al. 2003).

### Timing and duration of exposure measures

Questions about when, where, and how often to measure exposure are inherent in each of the prioritization schemes presented. Environmental exposures are likely to change over time and over space; thus, repeated measurements of exposure are desired. In this case, the timing, location, and duration of each sample within the life course and the microenvironments occupied by the subject will have to be determined.

It is crucial to consider the biologically relevant time scales of exposure. Is the exposure of interest short and intense or longer term and cumulative? And when is the relevant exposure period (window of susceptibility) in the continuum of development? Beyond this, the physicochemical properties of the exposure and the toxicokinetics must be considered. If biomarkers of exposure are being measured, then the relevant metabolites and half-life of the compound within the body must be known so that samples are collected at times that will accurately assess the exposure period of interest. If these values are unknown in humans, extrapolation from animal studies may be necessary, while recognizing that the dose and route of exposure in animal studies may not be the same for human environmental exposures.

In the case of studies on the development of childhood asthma, the outcome is a chronic (but in some cases transient or recurrent) disease. Depending on the exposure, we may be interested in either short-term peak exposures or in long-term chronic exposures occurring in the prenatal, perinatal, or postnatal period. Increased understanding of mechanisms, gene–environment interactions, and critical windows of exposure will facilitate decisions regarding the specific exposures and approaches for assessment.

Determination of the correct time to measure exposure is often impossible with the current state of knowledge. Information generated by birth cohort studies, including mechanisms of effect, may help determine when important windows of exposure occur. The need for plausible biological mechanisms must be highlighted. The biological target of the exposure must be considered, and the relevant developmental trajectory of the target organ or system must be at least partially understood. Without this knowledge, sample collection is more likely to be haphazard.

Environmental exposures from conception through the prenatal and perinatal periods and into childhood have been linked to the development of childhood asthma; thus the decision on when to measure exposure is difficult. Environmental exposures during pregnancy are important, but exposures during the first and second year of life are currently considered to be the most important (Dietert and Zelikoff 2008; Holt and Jones 2000; Peden 2000). This perspective may change as understanding of how epigenetic mechanisms impact the development of asthma deepens.

In relation to asthma, the development of both the respiratory and immune systems is relevant. These systems begin to develop in the early prenatal period and continue through the perinatal and postnatal periods (Becklake and Kauffmann 1999; Dietert and Zelikoff 2008; Holt and Jones 2000; Peden 2000; Pinkerton and Joad 2000; Pirruccello et al. 1989). It is possible that there are multiple windows of exposure, which may necessitate collection of multiple samples over the course of development. Further complicating this is the interaction between some exposures, such as endotoxin, and immune system development. This may mean that for the same compound there may be a window of protection as well as susceptibility in asthma (von Mutius 2007; von Mutius and Radon 2008).

### Methodologic advances in exposure assessment

Individuals are exposed to many xenobiotic substances in the course of daily life. The challenge for epidemiology is to separate the effects of different exposures and the contribution of one’s genetic profile.

One potential strategy to overcome these challenges is to assign exposure profiles to an individual based on their exposure to a combination of agents. First, a group of exposures (the exposure profile) is linked to the outcome; the next step determines which of the exposures in the profile is dominant. This is similar to risk stratification strategies in cardiovascular and other chronic diseases (Cannon and Greenberg 2008; Sabir et al. 2008). We often use composite exposures such as environmental tobacco smoke and traffic pollution to define complex mixtures. The agents that make up these mixtures are highly correlated, and it often makes more sense to use them as a composite rather than a set of separate but related exposures. This is similar to many risk factors for asthma that can be highly correlated in some populations.

In 2005, Wild published an editorial encouraging the development of an exposome to complement the genome (Wild 2005). The exposome would “encompass life course environmental exposures (including lifestyle factors) from the prenatal period onwards” and would evolve over the life course. The characterization of the exposome of an individual would be dependent on biomarkers (of exposure and/or effect) as well as improved questionnaire-based methods for assessing exposure. Detailed characterization of exposomes and subsequent linkage to different asthma phenotypes would theoretically aid in identification of groups of genes responsive to an exposure and groups of exposures that act through common pathways. Clearly, a considerable amount of detailed exposure data on large numbers of individuals would need to be compiled to begin to assess the extent to which a manageable number of unique exposomes exists. Optimally, such an effort should take place within the context of a cohort. In the long term, this approach may facilitate exposure profiling, leading to more effective and targeted preventative measures or treatments.

To sample exposure more frequently at minimal cost, participants can be engaged in data collection. This technique has been demonstrated using vacuum socks (Schram-Bijkerk et al. 2006), electrostatic wipes (Arbes et al. 2005; Cozen et al. 2008; Schram-Bijkerk et al. 2006), and passive samplers (Johnson et al. 2009). New methods for exposure measurement by subjects have also been proposed (Karlsson et al. 2002; Noss et al. 2008; Sercombe et al. 2005). However, there is a limit to subject participation in collecting exposure samples; subjects are unlikely to undertake a complicated or time-consuming protocol, and ethical issues surrounding subject burden may also need to be addressed.

The collection of additional samples, or additional sample volume for archiving, permits the future study of hypotheses that were not feasible or not yet relevant during the cohort planning stages. Often these analyses will take the form of nested case–control studies within the larger cohort. The collection of additional samples requires storage methods that preserve the integrity of samples over the entire storage period. The maximum duration of storage and ideal storage environment will depend on the type of sample (e.g., dust, blood) as well as the analyte of interest (e.g., endotoxin, hormones) (Douwes et al. 1995; Holl et al. 2008; Macher 2001).

### Ethical considerations

In addition to the ethical considerations inherent in all cohort studies, when exposure measurements are made in private residences, the findings can have serious implications for property values and occupant health. In the case of critical problems, there is responsibility to report the issue to the relevant authority immediately (i.e., child abuse or neglect). More generally, there is a duty to report back to the homeowner the findings of the investigation (Lambert et al. 2003; Paulson 2006). The homeowner has the right to understand the meaning of the results and how they affect both the occupants and the structure itself. For these reasons it is important to have a plan to communicate results to participants in a meaningful way. This could include both visual and written interpretations of the results, and the summary could be generic for each quartile (for example) of the data rather than specific to each home.

## Conclusions

Ideally, environmental samples would be obtained using accurate and precise methods during the relevant windows of exposure and processed appropriately to permit prompt analysis for existing hypotheses and the pursuit of additional research questions in the future. In reality, decisions are made based on available resources, the research interest of the individual researchers, and priorities of the funding organizations. A single study cannot do everything. Recognizing these limitations, there remains a significant need for better exposure assessment in epidemiological studies.

Exposure levels will vary between individuals and also over time and space within individuals. We must gain more knowledge of the biological mechanisms underlying disease to properly identify the windows of exposure, the relevant duration of exposure, and possible latency period(s). Multiple measures of exposure must be collected where possible. Modeling of exposures as well as subject participation in data collection may ease the burden of measuring exposure more frequently, but both require evaluation.

Researchers have to make decisions about what to measure, which microenvironment to measure in, where exactly to measure within each microenvironment, which methods to employ, and at what time point(s) to measure. These decisions involve tradeoffs between the accuracy and precision of exposure measures, the completeness of the exposure assessment, and the statistical power of the study, as well as the resources available and burden that will be placed on participants. Hypotheses about relevant exposures need to be based on knowledge of biological mechanisms, gene–environment interactions, and physiological development. Exposures of interest can be prioritized based on many levels, including public health importance, measurement ability, biological mechanism of action, and the future use of the data collected.

Gene–environment interaction studies are an area of immense research interest but are at even higher risk of being underpowered. Better exposure assessment and more precise phenotype ascertainment are the most efficient ways to increase the power in such studies. Secondarily, data pooling can also increase power, permit analyses not possible with smaller sample sizes, and increase the probability of replicable results.

Additionally, there is a need to consider data compatibility across cohorts when designing a new study. Exposures should be prioritized not only on the goals of the individual study but also on the power that may result from pooling data from multiple studies.

## Summary

Several areas of exposure assessment can be targeted for immediate improvement in studies of childhood asthma:

- Planning for data pooling, as well as storage of samples for future analyses, at the outset of cohort studies;
- Refining the exposure assessment protocol, questionnaires, and/or measurements to account for relevant time periods of exposure;
- Engaging participants in the measurement of exposure to permit the collection of multiple samples over the course of the study;
- Increased use of hybrid methodologies that combine pollutant data with atmospheric fate and transport, exposure, and dose models.

Ultimately, exposure is best characterized through a combination of information sources, and future studies should strive to improve their exposure-assessment strategy. Collectively, this information may yield unique exposure profiles common among subsets of the population that will help characterize children at risk and improve health outcomes.

## Figures and Tables

**Table 1:** Summary of exposure assessment in some previous birth cohort studies of asthma and allergies in children that did not focus exclusively on air pollution.

Cohort (references)	Year(s)	Type	Exposures	Sample type	Sample location	Timing of measurement
CaPPS (Canadian Asthma Primary Prevention Study) (Canada) (Becker et al. 1999, 2004; Carlsten et al. 2009)	1995	C, I, HR	HDM, cat (μg/g dust)	Dust	Bedroom floor (child, parent), mattress (child, parent), living room floor, furniture	Before birth, 2 weeks, 4 months, 8 months, 12 months, 18 months, 2 years, 7 years
			Dog (μg/g dust)	Dust		1 year, 2 years, 7 years
			ETS (ng/mg creatinine)	Biomarker	Urine (cotinine)	2 weeks
			ETS (ng/mg creatinine)	Biomarker	Breast milk (cotinine)	4 weeks
			Outdoor air pollution (NO, NO_2_, PM_2.5_, black carbon – μg/m^3^)	LUR model	Home address	1 year, 7 years
BAMSE (Sweden) (Almqvist et al. 2003; Emenius et al. 2003, 2004; Wickman et al. 2002)	1994–1996	C	Air change rate (changes per hour)	Measurement, passive tracer gas	All rooms	First winter season after birth
			Temperature (°F and °C)	Measurement	Living room and bedroom (child)	
			RH (g/kg)	Measurement	Living room and bedroom (child)	
			Indoor NO_2_ (μg/m^3^)	Air, passive sampler	Living room, outside living room window	
			Cat, dog (μg/m^3^ dust)	Dust, vacuum	Mattress (parent)	2 months
			Outdoor NO_2_ (μg/m^3^ dust)	Dispersion model using emission data	Home address(es)	First year of life
MAS (Multicentre Allergy Study) (Germany) (Lau et al. 2000, 2005; Nickel et al. 2002)	1990	C, HR	HDM, cat (ng/g dust)	Dust, vacuum	Living room, bedroom (child, parent)	6 months, 18 months, 3 years, 4 years, 5 years, 7 years
			HDM, cat (ng/g dust)	Dust, vacuum	Mattress	5 years, 10 years
			Endotoxin (EU/mg dust)	Dust	Mattress	10 years
PIAMA (Prevention and Incidence of Asthma and Mite Allergy) (Netherlands) (Brunekreef et al. 2000; Gehring et al. 2009; van Strien et al. 2000, 2002)	1996–1997	C, I, HR	HDM (ng/m^2^), cat (mU/m^2^), dog (dog ng/m^2^)	Dust, vacuum	Mattress (child, parent), living room floor	3 months, 4 years, 6 years, 8 years
			LPS (U/mg), EPS (U/mg), β-glucans (g/mg dust)	Dust, vacuum	Mattress (child), living room floor	3 months
			ETS (μg/m^3^)	Air (nicotine)	Home	2-week samples, 1997–1998
			Outdoor air pollution [PM_2.5_ (μg/m^3^), NO_2_ (μg/m^3^), soot (10^–5^ m^–1^)]	LUR model	Address at birth	Long-term average
MAAS (Manchester Asthma and Allergy Study) (United Kingdom) (Custovic et al. 2000)	1995–1997	C, I	HDM (ng/m^2^ and μg/g dust)	Dust, vacuum	Bed (child, parent), bedroom floor (child, parent), living room floor, furniture	Week 10 of pregnancy, birth, 6 months, 1 year
Boston, MA (USA) (Chew et al. 1998; Gold et al. 1999)	1994–1996	C, HR	HDM (μg/g), cat (μg/g), cockroach (μg/g)	Dust, vacuum	Bedroom floor (child), bed (child, parent), kitchen floor, chair/sofa	2–3 months
			Temperature (°C) and RH (absolute g/kg, relative %)	Measurement	Bedroom floor (child)	2–3 months
British (United Kingdom) (Arshad et al. 1992)	1990–1991	C, I	HDM (μg/g dust)	Dust, vacuum	Bedroom (child), living room floor, furniture	Birth, 3 months, 6 months, 9 months
PREVASC (Prevention of Asthma in Children) (Netherlands) (Kuiper et al. 2005; Schonberger et al. 2005a, 2005b)	1997–2002	C, I	HDM, cat, dog (ng/g dust and ng/m^2^)	Dust, vacuum	Mattress (child, parent), LR floor	3–5 months, 7–9 months, 4 years
			RH (NR)	Measurement	Bedroom (child, parent)	Months 3–5 and 7–8 of pregnancy, 4 weeks, 7–9 months, 1 year, 2 years, 4 years
			ETS (NR)	Biomarker	Exhaled carbon monoxide	
Krakow (Poland) (Jedrychowski et al. 2009)	2000–2003	C	ETS (ng/mL)	Biomarker	Cord blood (cotinine)	At birth
Detroit, MI (USA) (Ownby et al. 2002; Peterson et al. 1997, 1999)	1987–1989	C	ETS (ng cotinine/mg creatinine)	Biomarker	Urine (cotinine)	Every 2 months after birth for 2 years
			HDM (μg/g dust), cat (mU/g dust)	Dust, vacuum	Beside bed (child)	2 years
			HDM (μg/g dust), cat (mU/g dust)	Air	Bedroom (child)	2 years
Oslo (Norway) (Magnus et al. 1998; Nafstad et al. 1998)	1992–1993	C, CC	NO_2_ (μg/m^3^)	Air, passive sampler	Breathing zone (child); area: bedroom (child), kitchen wall, living room, child care, outside of house	During first 2 years of life, after meeting case/control definition
			HDM (count per bed)	Dust, vacuum	Mattress (child)	
			RH (g/kg)	Measurement	Living room	
			Air change rate (changes per hour)	Measurement, passive tracer gas	Whole home	

Abbreviations: C, cohort; CC, case control; EPS, extracellular polysaccharides (specifically from genera Penicillium and Aspergillus in PIAMA cohort); ETS, environmental tobacco smoke; HDM, house dust mites; HR, high risk; I, intervention; LPS, lipopolysaccharide; NR, not reported; RH, relative humidity.
